# The relationship between regional pain with or without neuropathic symptoms and chronic widespread pain

**DOI:** 10.1097/j.pain.0000000000001568

**Published:** 2019-04-08

**Authors:** John McBeth, Matthew R. Mulvey, Amir Rashid, James Anderson, Katie Druce

**Affiliations:** a Versus Arthritis Centre for Epidemiology and the NIHR Manchester Musculoskeletal Biomedical Research Unit, Central Manchester University Hospitals NHS Foundation Trust; The University of Manchester, Manchester, United Kingdom; b Academic Unit of Palliative Care, Leeds Institute of Health Research, University of Leeds, Leeds, United Kingdom

**Keywords:** Chronic widespread pain, Fibromyalgia, Neuropathic pain, Cohort study, Prospective, Population risk

## Abstract

This study was performed to test whether the risk of developing chronic widespread pain (CWP) in those with regional pain was augmented in those with symptoms of neuropathic pain (NP). Persons free of CWP completed the Douleur Neuropathique 4 (scores ≥3 indicating NP); demographics; Hospital Anxiety and Depression scale; Pittsburgh Sleep Quality Index; and pain medications. Participants were classified as having no pain, regional pain with no symptoms of NP (), or regional pain with symptoms of NP (NP). At the 12-month follow-up, participants with CWP were identified. Logistic regression estimated the odds ratio, with 95% confidence intervals, of CWP in the  and NP groups compared with no pain, and NP compared with . Partial population attributable risks estimated the proportion of CWP attributable to baseline  or NP exposure. One thousand one hundred sixty-two participants completed the baseline DN4 and provided pain data at follow-up: 523 (45.0%) had no baseline pain, 562 (48.4%) , and 77 (6.6%) NP. One hundred fifty-three (13.2%) had CWP at 12 months: 19 (3.6%) no pain, 108 (19.2%) , and 26 (33.8%) NP.  (2.9 [1.9-4.3]) and NP (2.1 [1.1-4.0]) predicted CWP after adjusting for demographics, Hospital Anxiety and Depression scale, Pittsburgh Sleep Quality Index, and medications. The partial population attributable risk was 41.3% (25.2-54.0) for  and 6.0% (0.1-11.6) for NP. The NP group were not more likely to develop CWP when compared directly with  (1.5 [0.8-2.8]). Neuropathic pain was relatively rare and predicted a small number of new-onset CWP cases. Using these estimates, treatments targeting NP would at best prevent 6% of CWP cases.

## Abstract 2

Neuropathic pain was relatively rare and predicted a small number of chronic widespread pain cases. Using these estimates, treatments targeting neuropathic pain would at best prevent 6% of chronic widespread pain cases.

## 1. Introduction

Chronic widespread pain (CWP), the clinical hallmark of fibromyalgia,^47^ is a significant health burden: it affects 11% of the general population,^31^ is associated with higher disability,^44^ and increases mortality^30^ most likely through reduced physical function.^40^ Of the estimated 33% to 50%^7,44^ of the general population who have a painful regional musculoskeletal disorder (eg, back pain and knee pain), up to 20% develop CWP.^20,33,34^ Risk factors for CWP include sleep problems,^1^ childhood chronic physical illness,^37^ and trauma.^23^ In a systematic review, the transition from regional musculoskeletal pain to CWP was predicted by pain independently of sex, increasing age, family history of pain, and depression.^28^

Neuropathic pain (NP), pain distinguished from other pain conditions by characteristic symptoms such as “burning” or “freezing,”^11,14^ is present in up to 16% of persons with CWP,^35^ double the population prevalence of 8%.^15^ The heritability of NP and CWP is correlated and the genetic predisposition to NP shared with CWP.^35^ There are no prospective studies testing whether the risk of developing CWP in those with regional pain is augmented by the presence of NP.

This study tested the hypothesis that the risk of developing CWP would be augmented by the presence of pain with symptoms of NP.

## 2. Methods

In a prospective population-based cohort study (the Pain across the Adult Lifespan study^8^), participants were invited to complete a baseline questionnaire. Those free of CWP were identified and classified as having no pain, pain without neuropathic symptoms (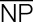), or NP. All participants were followed up 12 months later, at which time they completed a second questionnaire and those with CWP identified. The relationship between pain status at baseline and CWP at follow-up was examined.

Ethical approval was granted by the North West 8 Research Ethics Committee—Greater Manchester East (reference number 10/H1013/29).

### 2.1. Baseline questionnaire

The baseline questionnaire collected data on the presence, location, and duration of pain. Participant's reports of pain were used to identify those with prevalent CWP, classified using published criteria^13^ that have been used in previous population-based surveys^24^ (see below for details). The screening questionnaire also collected data on demographics (date of birth; sex; English Index of Multiple Deprivation 2007,^38^ with higher scores indicating less deprivation; Hospital Anxiety and Depression (HAD)^48^ depression and anxiety subscales (7 items each, scored 0-3, total score 0-21, higher scores represent higher probability of depression or anxiety); Pittsburgh Sleep Quality Index^10^ (19 items, scored 0-3, total score range 0-57); Rapid Assessment of Physical Activity^42^ (7 items, scored as sedentary, underactive, underactive regular, and active); self-reported pain medications (summed to give a total count); and signed consent.

### 2.2. Presence, distribution, and duration of musculoskeletal pain

The questionnaire included a detailed assessment of the presence of musculoskeletal pain. All participants were asked “During the past month, have you experienced any pain which has lasted at least 1 day or longer?” Respondents answering positively were asked to shade the location(s) of their pain on a four-view body manikin. Participants were then asked whether they had been aware of their pain for 3 months or longer (yes/no). Using their responses to these questions, participants were classified as having CWP using the definition in the American College of Rheumatology (ACR) 1990 classification criteria for fibromyalgia^47^ that was operationalised by Croft et al.^13^ as requiring axial (cervical spine, thoracic spine, anterior chest, or low back) pain and pain in contralateral body quadrants, above and below the waist, and on the right and left hand sides of the body, which has been present for at least 3 months. Remaining participants were classified as having some pain (those participants reporting pain that did not satisfy the criteria for CWP) or no pain. The data collection tool (a blank body manikin) has been shown to collect valid and reliable pain data with high between-rater reliability scoring.^27^ The original criteria for CWP defined by Croft et al.^13^ were based on the definition included in the 1990 classification criteria for fibromyalgia that required pain in the axial skeleton and in contralateral body quadrants. The criteria have been used in multiple population-based epidemiological studies and have face and construct validity (they identify persons with high care utilisation, poor sleep, depression, physical inactivity, and with higher tender point counts among many other findings)^9,26^ and have been shown to identify persons with a genetic susceptibility to developing CWP.^29,39^

### 2.3. Neuropathic pain

Participants reporting the presence of pain were invited to report the sensory characteristics of their pain symptoms using the Douleur Neuropathique 4 (DN4) questionnaire.^5^ Participants were asked “Thinking about your single worst pain, does the pain have one or more of the following characteristics?”; burning, painful cold, electric shocks, tingling, pins and needles, numbness, and itching. For each characteristic responses were scored yes (=1) or no (=0). Participants scoring 3 or more were considered to have pain of predominantly neuropathic origin. The short version of the DN4 has been validated for use in self-reported questionnaires with 83% sensitivity and 90% specificity when compared to clinical diagnosis.^4^ The interrater reliability of the DN4 was demonstrated in the original French version with kappa values for individual items ranging from 0.66 to 0.96^5^ and has subsequently been demonstrated cross-culturally, although the level of supporting evidence is low.^32^

### 2.4. Follow-up questionnaire

A follow-up questionnaire was mailed to those baseline participants who were free of CWP, 12 months after the date they returned a baseline questionnaire. The follow-up questionnaire included identical methods to assess pain as those used in the baseline survey. Based on their pain reports at follow-up, participants were classified as “CWP” for those who reported pain that satisfied the criteria for CWP or “Not CWP.”

### 2.5. Analysis

Logistic regression estimated the odds of CWP first in the 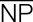 and NP groups compared with the no pain group (the referent group), second in the NP group compared with the 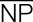 group (the referent group), and finally for the individual pain characteristics with those participants not reporting the characteristic being classified as the referent group. Results were expressed as odds ratios (ORs) with 95% confidence intervals (CIs). For those comparisons with significant ORs, population attributable risks (PARs) were calculated using formulae for cohort studies.^21,41^ These estimated the % of CWP that would be avoided if participants were not exposed to 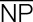 or NP. In an evaluation of a preventive intervention in a multifactorial disease setting, the interest is in the percent of cases associated with the exposures to be modified, when other risk factors, possibly nonmodifiable, exist but do not change as a result of the intervention. The partial population attributable risk (PARp) was proposed to estimate this quantity. Under the assumption of no interaction of the index exposure effects with the background risk factors, the PARp is formulated as
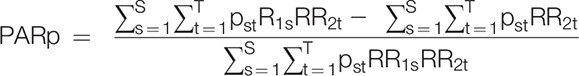

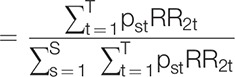
where t denotes a stratum of unique combinations of levels of all background risk factors which are not under study, t = 1; …; T and RR_2t_ is the relative risk in combination t relative to the lowest risk level, where RR_2,1_ = 1. S indicates an index exposure group defined by each of the unique combinations of the levels of the index risk factors, that is, those risk factors to which the PARp applies, s = 1; …; S, and RR_1s_ is the relative risk corresponding to combinations relative to the lowest risk combination, RR_1,1_ = 1. The joint prevalence of exposure group s and stratum t is denoted by p_st_, and p_.t_ = 
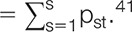
.^41^

## 3. Results

### 3.1. Participation rates

**Figure 1.**
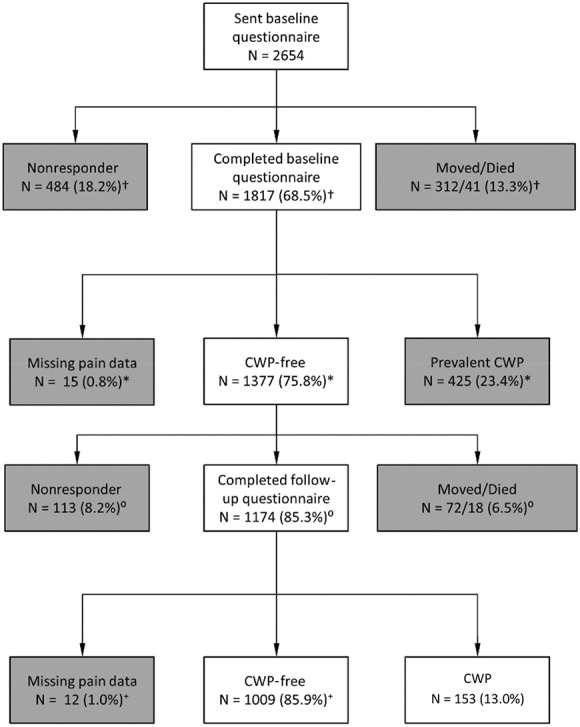
Flow diagram of participant recruitment and CWP rates. Primary care records were reviewed to identify those who were recorded as either having moved address or had died after the questionnaire mailing. CWP-free = CWP-free at baseline and follow-up; CWP = CWP-free at baseline and CWP at follow-up; †Proportions are of participants who sent baseline questionnaire. *Proportions are of participants who returned a baseline questionnaire. ⁰Proportions are of participants free of CWP at baseline. ⁺Proportions are of participants free of CWP at baseline and mailed a follow-up questionnaire. CWP, chronic widespread pain.

Of 2654 persons who were mailed a baseline questionnaire, 1817 responded (68.5%, adjusted for 312 [11.8%] participants who had moved from their recorded address and 41 [1.5%] who had died) (Fig. 1). One thousand three hundred seventy-seven (75.8%) participants were free of CWP and were mailed a follow-up questionnaire, and 1174 (85.3%, adjusted for those who had moved or died) responded.

### 3.2. Rate of chronic widespread pain and associated factors

Of the 1174 participants at follow-up, 12 (1.0%) were missing pain data, leaving 1162 participants for analysis. Of the 1162 participants, 523 (45.0%) had no pain, 562 (48.4%) had 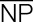, and 77 (6.6%) had NP at baseline (Table 1). The proportion of participants classified as CWP at follow-up was significantly higher among those with 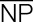 (19.2%) and those with NP (33.8%), when compared with the proportion in those reporting no pain at baseline (3.6%). Baseline reports of NP characteristics assessed in the DN4 questionnaire were more common in those with CWP at follow-up (*P* < 0.001 for all comparisons). Chronic widespread pain was associated with having significantly more pain sites at baseline, higher pain medication use at baseline, female sex, and higher HAD anxiety, HAD depression, and sleep problem scores at baseline. There was no association with age, occupational status, or deprivation score (Table 1).

**Table 1:**
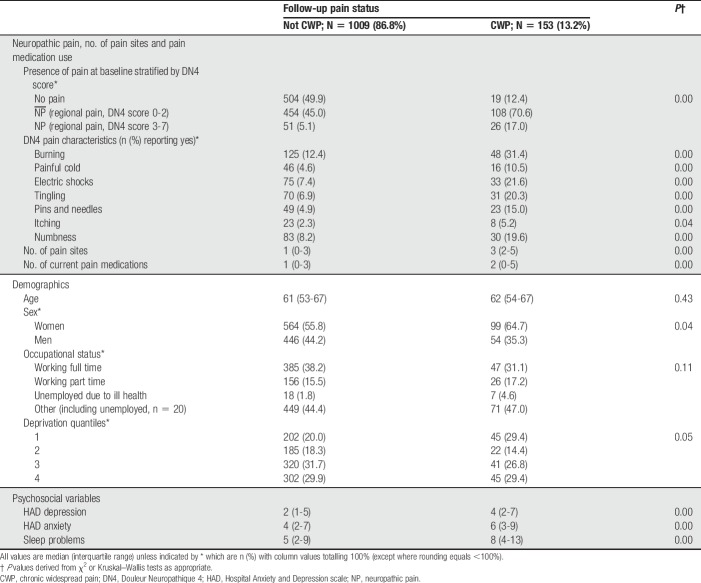
Participant characteristics at baseline stratified by chronic widespread pain status at follow-up.

### 3.3. The relationship between neuropathic pain and chronic widespread pain

When compared to those with no pain at baseline, after adjusting for age and sex (Table 2), 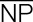 was associated with a 3-fold (OR = 2.9, 95% CI [2.0-4.2]) increased odds of CWP at follow-up, and NP was associated with a 4-fold (3.9 [2.3-6.4]) increased odds. After adjusting for age, sex, psychosocial variables, demographics, and medication use, the association between 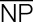 and CWP persisted (2.9 [1.9, 4.3]), whereas the association between NP and CWP was attenuated (2.1 [1.1-4.0]). After adjusting for all covariates, the NP characteristics of burning, electric shocks, tingling, pins and needles, and numbness were all significantly associated with CWP.

**Table 2:**
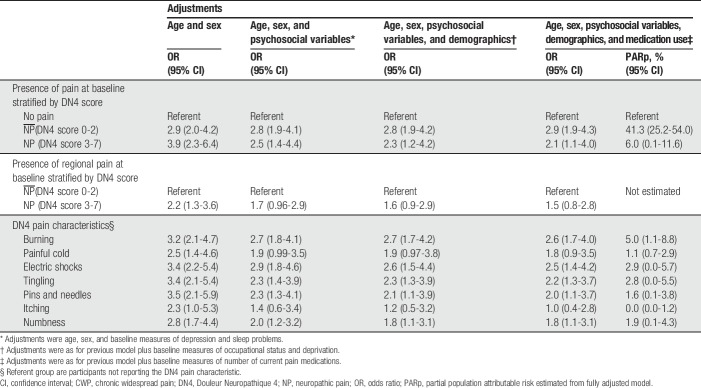
Regional pain stratified by DN4 score and DN4 pain characteristics and CWP: results from multinomial logistic regression models.

After adjusting for age and sex, those with NP were twice (2.2 [1.3-3.6]) as likely to have CWP at follow-up when compared with the 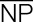 group (Table 2). However, the relationship between NP and CWP was attenuated and no longer significant (1.5 [0.8-2.8]) when adjusted for age, sex, psychosocial variables, demographics, and medication use.

### 3.4. Partial population attributable risks

The proportion of all cases of CWP that could be attributed to the presence of NP at baseline was 6.0% (95% CI [0.1-11.6]), whereas 41.3% (25.2, 54.0) could be attributed to the presence of 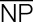 (Table 2). The proportion of CWP attributable to the NP characteristics ranged from 1.9% to 5.0% (Table 2).

## 4. Discussion

### 4.1. Summary of findings

This study tested the hypothesis that the relationship between regional pain and CWP would be augmented by the presence of symptoms of NP. The data presented here do not support that hypothesis. When compared to those persons with no pain, having some pain was associated with an increased odds of developing CWP. The relationship with CWP initially seemed to be stronger among those persons with symptoms of NP, although the effect was attenuated when adjusted for age, sex, occupational status, deprivation, anxiety, depression, and sleep problems to the extent that 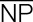 had a stronger relationship with CWP. The data suggest that, at a population level, the proportion of all new cases of CWP that could be attributed to NP was modest, and was lower than the proportion attributable to 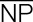.

### 4.2. Setting these results in the context of current literature

The data presented here show that having regional pain increases the odds of CWP by 2-fold, irrespective of the presence of NP symptoms. Previous longitudinal studies have shown similar strengths of associations with arm pain (relative risk [RR] 2.4 [1.5-4.3]) and back pain (RR 2.5 [1.5-6.7])^17^ and pain at multiple sites (OR 2.6 [1.3-5.4])^22^ with CWP. In contrast to the findings presented here, some previous cross-sectional studies have supported the role of NP in CWP and associated disorders. Fishbain et al.^16^ reported that chronic pain patients including those with fibromyalgia scored above the cutoff on the Neuropathic Pain Scale. Fibromyalgia patients commonly report sensory symptoms characteristic of NP with 83% reporting deep-tissue hyperalgesia, 51% reporting burning, and 47% reporting prickling on the painDETECT questionnaire.^2,18^ There is suggestive evidence for an overlap between NP and CWP or fibromyalgia; these disorders share common risk factors including age, sex, body mass index, smoking, and socioeconomic status.^35^ However, these associations are not consistent: patients with painful diabetic neuropathy when compared to those with fibromyalgia were less likely to be female, had a higher BMI, were less likely to be depressed or anxious, and had better sleep patterns.^25^

Fibromyalgia and CWP are clearly related, with fibromyalgia being a subset of persons with CWP.^46^ However, fibromyalgia is not CWP, and one of the main differences may be the presence of NP. In a study of twins, 15% of those with CWP had features of NP, the heritability of NP and CWP were correlated, and the genetic predisposition to NP was shared with that of CWP.^35^ These associations may be driven by the subset of twins who had fibromyalgia, and this is supported by the findings of similar results when the data set was restricted to the subgroup of twins with fibromyalgia.^35^ The data presented here support the role of regional pain in the onset of CWP and clearly show that the relationship is independent of the presence of NP symptoms. It is possible that some participants with asymptomatic (ie, not painful) small fibre neuropathy could be more likely to develop CWP; however, we did not collect that data and are unable to address that question in the current study.

### 4.3. Methodological strengths and limitations

This study has several strengths. The cohort was a large, unselected group of persons free of CWP at baseline. The study used validated assessments of pain, NP, and all covariates. Previous studies that were cross-sectional were unable to determine the temporal association between the presence of neuropathic features of acute localised pain and the transition to CWP. Here, participants who were free of CWP were identified and followed up over time to identify those who developed CWP, allowing the temporal relationship between having NP and developing CWP to be established. Previous studies have examined clinic populations where levels of psychological distress are higher than unselected general population samples, and high levels of psychological and emotional distress are known to be related to levels of pain reporting, somatosensory changes, and altered nociceptive processing.^45^ Limitations were that participants were followed up once, 12 months after they completed their baseline questionnaire, and it was not clear what happened in the intervening months between baseline and follow-up. Although those reporting CWP at follow-up were identified and classified as the CWP group, it is likely that some participants may have developed CWP that then resolved before follow-up. We would have missed those incident cases, underestimated the rate of CWP in this cohort, and misclassified participants as Not CWP instead of CWP. How would that impact on the associations reported here? If the misclassification was random across baseline exposure status (ie, was independent of baseline pain status) it could act to attenuate the observed association and we may have underestimated the association between NP and the onset of CWP. Any underestimation of effect is unlikely to impact significantly on the pPAR estimates here because, although the PAR is based on the effect estimate, it is heavily influenced by how common a risk factor is in the population. Among those free of CWP, baseline 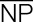 was much more common, with 48.4% of participants having had 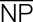 and 6.6% having NP.

Several screening tools have been developed to assess NP characteristics. Previous studies using the DN4 and LANSS have estimated the prevalence of pain of predominately neuropathic origin to be between 7% and 8% of the general population.^6,43^ However, the use of NP screening tools in self-reported surveys of musculoskeletal pain is contentious because not all pains are the same.^36^ NP screening tools have been primarily validated for use in subjects with single-site pain disorders (such as low back pain, diabetic neuropathy, or trigeminal neuralgia) and require the respondent to focus on a single painful region or area.^3,5,18^ Although the participants in this study did not have CWP at baseline, CWP by its nature is multisite and widespread, and the suitability of these screening tools for use in studies of multisite pain conditions is not clear.^36^ A recent epidemiological survey of NP symptoms in the general population navigated this issue by asking participants to indicate the site(s) of any chronic pain on a body map and then to identify the most troublesome site of pain from a list of body regions, and subsequent questions on neuropathic symptoms were focused on this “most troublesome pain.”^43^ That is, the method we used here, and although this method is not without its shortcomings, it does allow for the assessment of NP symptoms in population-based surveys of musculoskeletal pain which would otherwise not be possible.

### 4.4. Clinical implications

The effectiveness of pharmacological treatments for NP in patients with CWP disorders such as fibromyalgia are not clear with systematic reviews showing the supporting evidence for gabapentin is weak,^12^ and there are no high-quality trials of oxycodone.^19^ In this study, NP was relatively rare and predicted a small number of new-onset CWP cases. Using these estimates, treatments targeting NP would at best prevent 6% of CWP cases. Chronic widespread pain is highly prevalent in the general population, and effective treatment of pain not of NP origin will have the biggest impact on population levels of CWP.

## Conflict of interest statement

The authors have no conflict of interest to declare.
